# Quantitative diagnosis of breast tumors by morphometric classification of microenvironmental myoepithelial cells using a machine learning approach

**DOI:** 10.1038/srep46732

**Published:** 2017-04-25

**Authors:** Yoichiro Yamamoto, Akira Saito, Ayako Tateishi, Hisashi Shimojo, Hiroyuki Kanno, Shinichi Tsuchiya, Ken-ichi Ito, Eric Cosatto, Hans Peter Graf, Rodrigo R. Moraleda, Roland Eils, Niels Grabe

**Affiliations:** 1 Department of Pathology, Shinshu University School of Medicine, Nagano, Japan; 2 Division of Theoretical Bioinformatics, German Cancer Research Center (DKFZ), Heidelberg, Germany; 3 Department for Bioinformatics and Functional Genomics, Institute of Pharmacy and Molecular Biotechnology (IPMB) and Bioquant, University of Heidelberg, Heidelberg, Germany; 4 RIKEN Center for Advanced Intelligence Project, Pathology Informatics Unit, Tokyo, Japan; 5 Quantitative Pathology & Immunology, Tokyo Medical University, Shinjuku, Tokyo, Japan; 6 Department of Molecular Pathology, Tokyo Medical University, Shinjuku, Tokyo, Japan; 7 Diagnostic Pathology, Ritsuzankai Iida Hospital, Nagano, Japan; 8 Division of Breast and Endocrine Surgery, Shinshu University School of Medicine, Nagano, Japan; 9 Department of Machine Learning, NEC Laboratories America, NJ, USA; 10 Applied Tumor Immunity Clinical Cooperation Unit, National Center for Tumor Diseases, German Cancer Research Center, Heidelberg, Germany; 11 Department of informatics, Technical University Federico Santa Maria Valparaiso, Chile; 12 Department of Medical Oncology, National Center for Tumor Diseases, University of Heidelberg, Heidelberg, Germany; 13 Hamamatsu Tissue Imaging and Analysis Center, Bioquant, University of Heidelberg, Heidelberg, Germany

## Abstract

Machine learning systems have recently received increased attention for their broad applications in several fields. In this study, we show for the first time that histological types of breast tumors can be classified using subtle morphological differences of microenvironmental myoepithelial cell nuclei without any direct information about neoplastic tumor cells. We quantitatively measured 11661 nuclei on the four histological types: normal cases, usual ductal hyperplasia and low/high grade ductal carcinoma *in situ* (DCIS). Using a machine learning system, we succeeded in classifying the four histological types with 90.9% accuracy. Electron microscopy observations suggested that the activity of typical myoepithelial cells in DCIS was lowered. Through these observations as well as meta-analytic database analyses, we developed a paracrine cross-talk-based biological mechanism of DCIS progressing to invasive cancer. Our observations support novel approaches in clinical computational diagnostics as well as in therapy development against progression.

In several European countries and the United States, over 10 percent of women are diagnosed with breast cancer at some point during their lifetime^1^,^2^. Breast cancer is now a common disease of females in western countries. The watershed event in breast cancer is the progression from the pre-invasive stage of ductal carcinoma *in situ* (DCIS) to invasive cancer breaking through the basal membrane. However, not all DCIS will progress to invasive cancer during a woman’s lifetime^3^. Recently, micro-environmental cells, myoepithelial cells surrounding cancer cells, are studied whether they act as a barrier against cancer invasion in the breast^4^,^5^.

Histology slides of breast tissue contain many types of cells: luminal cells, myoepithelial cells, fibroblasts, lymphocytes, macrophages, etc. Myoepithelial cells are micro-environmental cells separating the luminal epithelial cells from the interstitial matrix of the basement membrane. Smooth muscle actin (SMA), CD10, calponin, cytokeratins 14 and 17 (CK14 and CK17), and p63 are known as markers of myoepithelial cells^6^,^7^. Myoepithelial cells play an important role as a physical barrier to cancer cell invasion and in producing the basement membrane^5^, expressing tumor suppressor proteins^8^,^9^,^10^ as well as antiangiogenic^11^ and antiproliferative factors^12^. On the other hand, it is also known that the phenotype of myoepithelial cells is sometimes altered in DCIS ducts^5^, where they change their secretion of tenascin-C isoform to a more fetal phenotype thereby promoting cell migration^13^. Between normal and DCIS tissues they experience dramatic gene expression changes through epigenetic alterations^14^,^15^. While many studies have analyzed these cells at the molecular level, so far only few have used quantitative morphological analysis.

Recent progress in digital pathology has demonstrated the power of quantitative image analysis for the study of pathological lesions^16^. Beck *et al*. developed the C-Path (Computational pathologist) system to measure a large number of features from breast cancer epithelium and stroma, and they found that stromal features were significantly associated with survival rates^17^. Dahlman *et al*. verified, using automated image analysis, that Beta-microseminoprotein was a strong factor in favorable outcomes after radical prostatectomy for localized prostate cancer^18^. Veta *et al*. automatically analyzed nuclear size of male breast cancer cells and found that the mean nuclear area proved to be a significant prognostic indicator^19^. Yuan *et al*. found that image processing allowed them to describe and validate an independent prognostic factor based on quantitative analysis of spatial patterns between stromal cells^20^. These studies succeeded in connecting subtle morphological changes of cells on pathology slides and prognostic factors of patients by using quantitative image analysis technologies. Furthermore, we have been involved into the development of “e-Pathologist”, a computer system that measures morphological features on pathology slides and classifies regions as normal or suspicious^21^,^22^. Such quantitative morphological studies allow to analyze subtle and complex interactions of measured features which often cannot be detected by the human eye.

In this study, we show that histological types of intraductal proliferative lesions can be classified by a machine learning system using only subtle morphological variations of myoepithelial cells and without any information about tumor cells. Furthermore, we propose a possible biological mechanism of these morphological changes and its clinical application.

## Materials and Methods

### Subject

We analyzed a total of 11661 myoepithelial cells in 22 cases (see Supplemental Table S1): 7 cases of normal breast tissue: age 65.4 ± 13.9 (mean ± SD), 5 cases of usual ductal hyperplasia (UDH): age 56.6 ± 7.6, 5 cases of low grade DCIS (LG-DCIS: DCIS G1): age 65.0 ± 15.6, 5 cases of high grade DCIS (HG-DCIS: DCIS G3): age 63.8 ± 11.6. There was no significant difference in age between each histologic type (t-test). Note that this study doesn’t include atypical ductal hyperplasia (ADH). ADH has a four to five times greater risk of complication into invasive cancer compared with UDH^23^,^24^. However, the biological underpinnings of ADH are controversial and therefore no ADH cases were analyzed in this study. All patients were diagnosed and operated in Shinshu University Hospital and none included an invasive lesion nor were they treated with neoadjuvant chemotherapy. No case of needle biopsy was included. This study was performed according to the Declaration of Helsinki and was approved by the ethical committee, Shinshu University, Japan. We obtained informed consent from all participants involved in our study.

### Histological classification of tumors

We classified tumors histologically based on the World Health Organization (WHO) classification criteria. At least 3 pathologists diagnosed and scored all cases independently and reached collective consensus.

### Immunohistochemical staining

Immunohistochemical staining was performed according to standard procedures. Briefly, all samples were fixed in 10% formalin and embedded in paraffin. The paraffin‑embedded tissue blocks were sectioned at a thickness of 4 μm. The sections were deparaffinized in xylene and rehydrated in ethanol solution. Antigen retrieval was performed in EDTA buffer (pH 8.0) using a microwave (30 min) and cooled to room temperature. Endogenous peroxidase activity was blocked by incubation in 3% H_2_O_2_ for 10 min. Following rinsing with wash buffer, sections were incubated for 1 hour with mouse monoclonal p63 antibody (abcam, Tokyo, Japan) at a dilution of 1:50. Subsequently, Histofine simple stain MAX-PO(M) (NICHIREI BIOSCIENCES, Tokyo, Japan) were used for detection. The sections were developed with Diaminobenzidine Tetrahydrochloride (DAB) (Dojin, Kumamoto, Japan) and counterstained with hematoxylin. Negative controls were performed by substituting the primary antibody with a nonimmune serum.

### Quantitative morphological image analysis

We used two types of stains on serial sections for each tissue. Using p63 immunohistochemistry, myoepithelial cell nuclei can be clearly detected, however intra-nuclear textures are not revealed. On the other hand, hematoxylin and eosin (HE) stained images require manual cell segmentation but reveal a wide spectrum of intra-nuclear textures. In addition HE stains also provide a basis for routine diagnostics by pathologists. We therefore performed comparative image analysis on both stains.

### p63 immunohistochemistry images analysis

**Figure 1:**
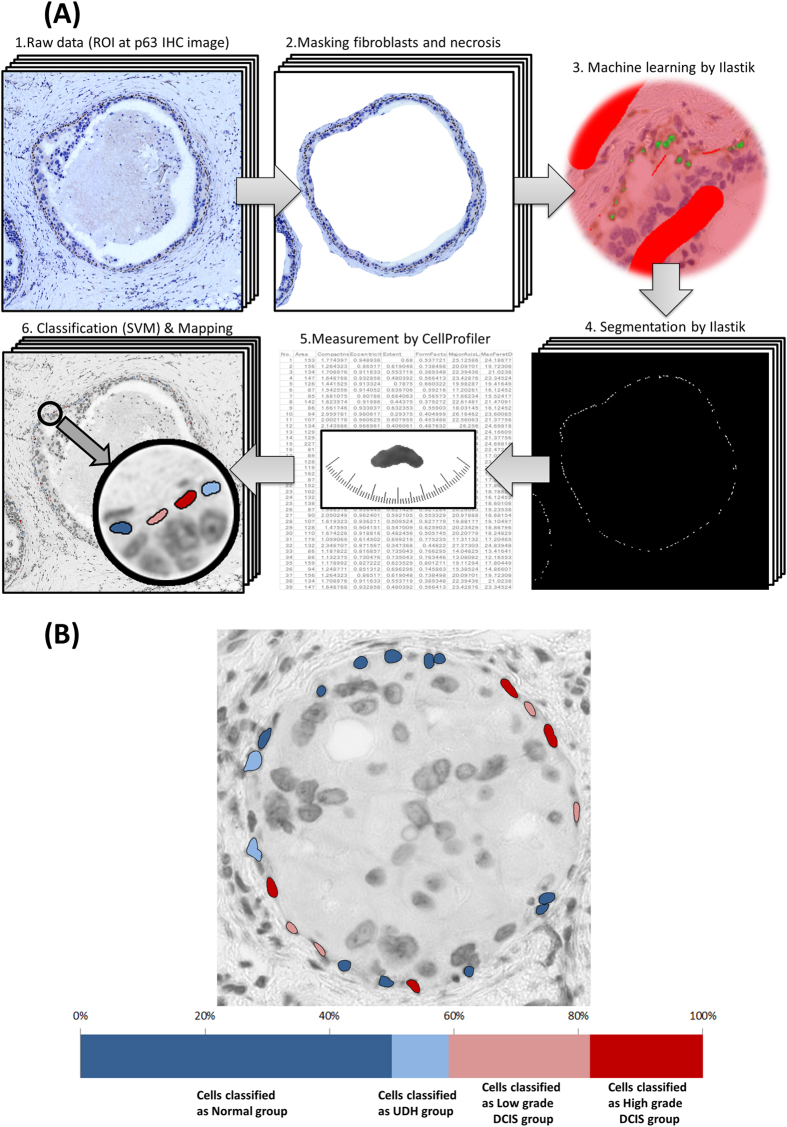
(**A**) Flowchart of image analysis on p63 immunohistochemistry images. 1 All slides were scanned by a whole slide image (WSI) scanner and a total of 70 ROIs were selected manually from p63 immunohistochemistry images. 2. To select only myoepithelial cell nuclei, we masked fibroblasts in interstitial tissue and inside the ducts as well as necrosis. 3. We applied Ilastik (segmentation software) to these ROIs. This is the training phase of machine learning for segmentation. 4. Segmentation by the trained Ilastik is applied to other images. 5. Each cell was measured using CellProfiler. 6. High dimensional morphological features of each cell were applied to machine learning, support vector machine (SVM). Based on the result of SVM classification, myoepithelial cell nuclei are drawn in different colors. **(B)** Example of heterogeneity of myoepithelial cells within a duct. Myoepithelial cells are marked by each histologic type based on classification of SVM: dark blue (myoepithelial cells classified as normal group), light blue (cells classified as UDH group), light red (cells classified as LG-DCIS group), dark red (cells classified as HG-DCIS group). A bar graph shows the proportion of each classified cell type in the duct.

**Figure 2:**
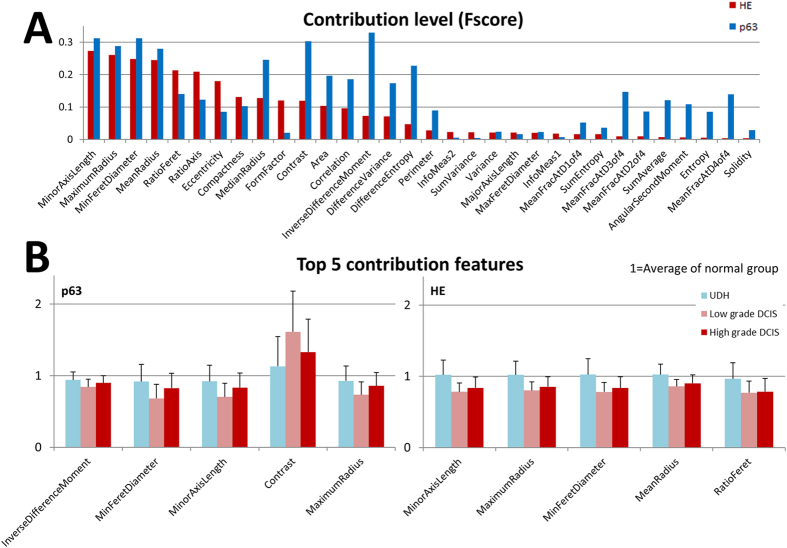
(**A**) Contribution level of each morphological feature. The F-score denotes the discrimination power of each individual feature. Red bar: F-score on the HE stained image, blue bar: F-score on the p63 immunohistochemistry images. (**B**) Top 5 contribution features. Ratio of top 5 contribution feature’s average value with standard deviation (1 = Average of normal group). Light blue (cells classified as UDH group), light red (cells classified as LG-DCIS group), dark red (cells classified as HG-DCIS group).

**Figure 3:**
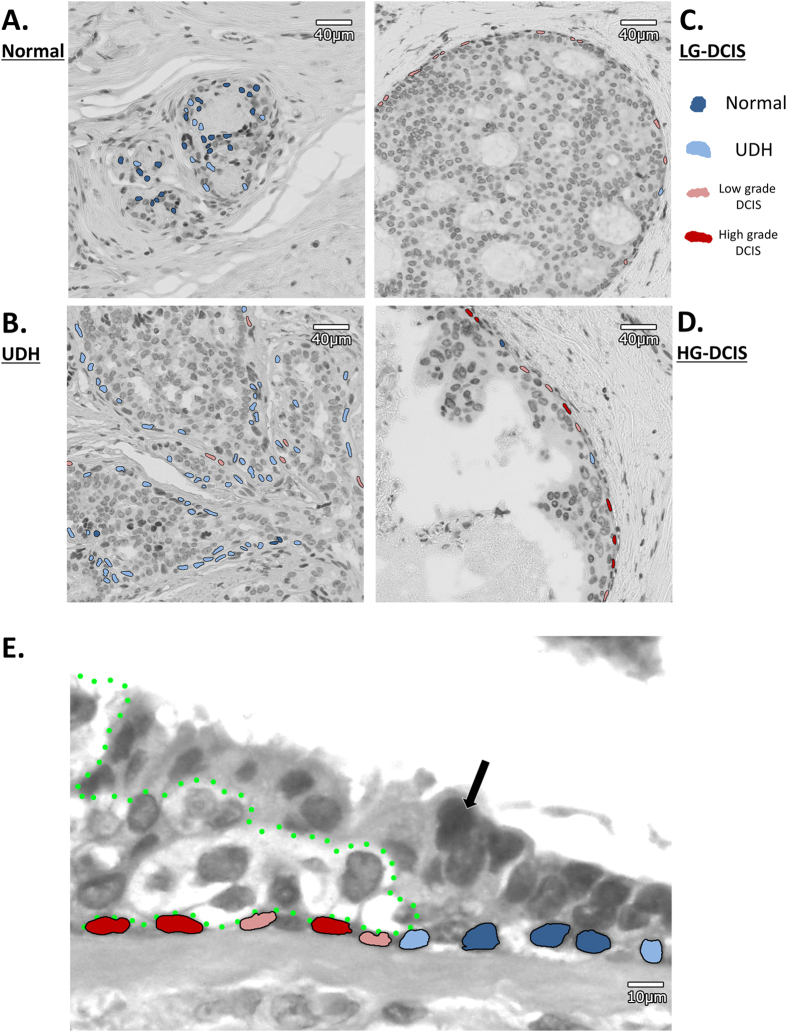
Representative examples of heterogeneity mapping of myoepithelial cells. (**A**) Normal area, (**B**) UDH area, (**C**) LG-DCIS area, (**D**) HG-DCIS area. (**E**) Edge area of DCIS. Neoplastic cells push through between normal luminal cells and myoepithelial cells. Black arrow: Normal epithelial cell. Cells surrounded by green dot line: neoplastic cells. Dark blue cells: myoepithelial cells classified as normal group, light blue cells: myoepithelial cells classified as UDH group, light red cells: myoepithelial cells classified as LG-DCIS group, dark red cells: myoepithelial cells classified as HG-DCIS group.

**Figure 4:**
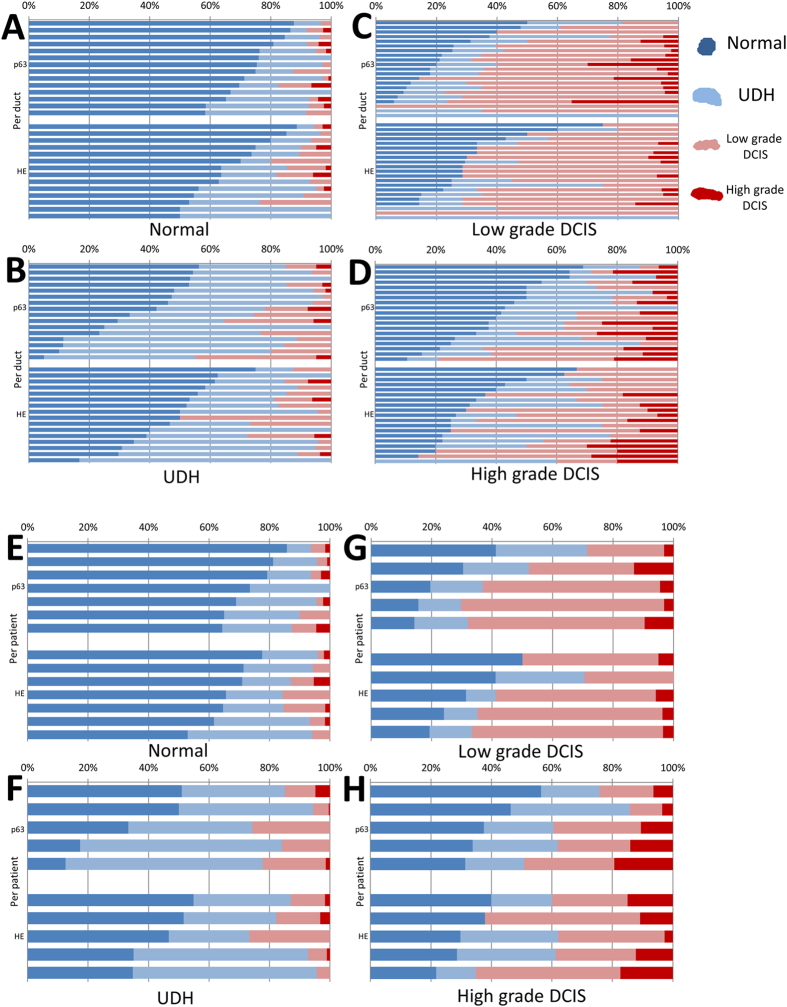
Heterogeneity analysis. Proportion of myoepithelial cells marked as each histologic type based on SVM classification. (**A**) Normal group on duct level, (**B**) UDH group on duct level, (**C**) LG-DCIS group on duct level, (**D**) HG-DCIS on duct level, (**E**) Normal group on patient level, (**F**) UDH group on patient level, (**G**) LG-DCIS group on patient level, (**H**) HG-DCIS on patient level. Dark blue (myoepithelial cells classified as normal group), light blue (cells classified as UDH group), light red (cells classified as LG-DCIS group), dark red (cells classified as HG-DCIS group).

**Figure 5:**
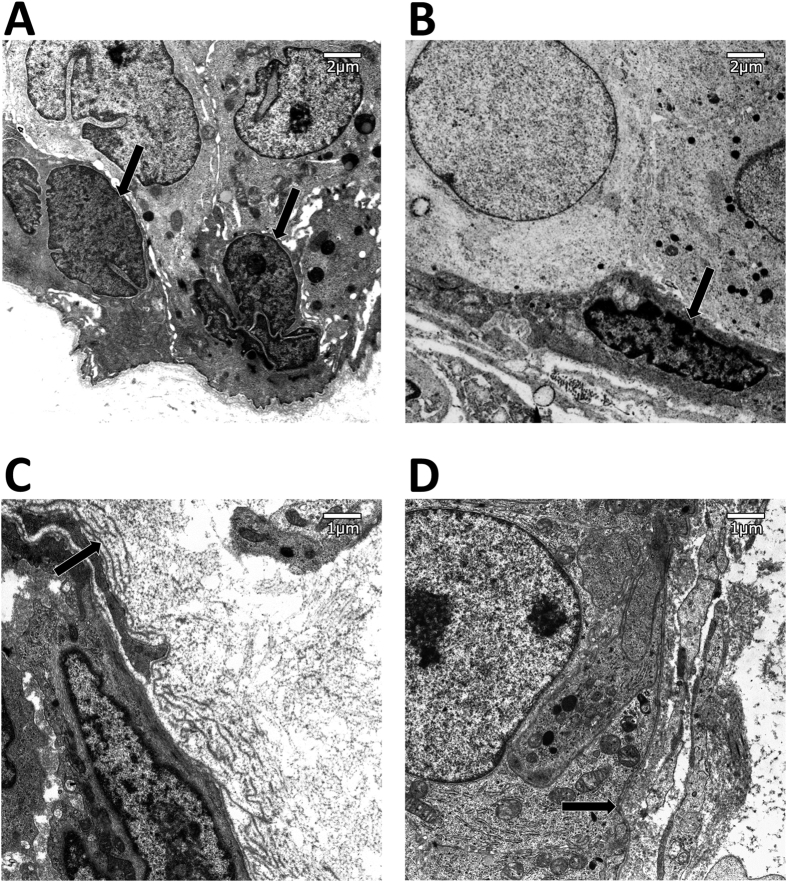
Electron micrograph of myoepithelial cell nucleus. Myoepithelial cells in DCIS lesions have a flatter nucleus than in UDH. Arrow: Myoepithelial cell nucleus. (**A**) Myoepithelial cell nucleus in a benign case. (**B**) Myoepithelial cell nucleus in a DCIS case. Electron micrograph of basal lamina. In benign ducts, the basal lamina has multiple layers, while in DCIS it has a single layer. Arrow: Basal lamina. (**C**) Basal lamina in a benign lesion. (**D**) Basal lamina in a DCIS lesion.

Figure 1A shows the flowchart of image analysis of a p63 immunohistochemistry image. All slides were scanned by the whole slide image (WSI) scanner (Hamamatsu NanoZoomer 2.0-HT Slide scanner) at 20x image magnification and images were stored on a computer system (Fig. 1A-1). In this paper, since we focused on analysis of pathological interest, a total of 70 ROIs (region of interest) were selected manually from p63 immunohistochemistry images. Supplemental Table S2 shows how these were distributed between the 22 cases or the 4 histological types. The size of a ROI is 2174 × 2174 pixels and corresponds to 1 mm^2^ of tissue. ROIs were stored in Tagged Image File Format (TIFF) for lossless compression. In order to analyze only myoepithelial cell nuclei, we manually masked fibroblast cells in interstitial tissue as well as necrotic tissue (Fig. 1A-2). After masking, segmentation of cell nuclei was performed by the system. For p63 immunohistochemistry images, we applied the Ilastik (version 1.1.3) segmentation software. Ilastik extracts myoepithelial nuclei using the random forest machine learning approach^25^. It was first trained using one DCIS ROI (Fig. 1A-3), and then applied to the remaining ROIs (Fig. 1A-4). To reduce noise due errors in nucleus contour extraction (for example two or more touching nuclei resulting in a single contour), we use statistics of features over 80% of the centered nuclei, where 10% of the largest nuclei as well as 10% of the smallest are ignored. Next, we measured each identified myoepithelial nucleus using CellProfiler^26^ (Fig. 1A-5). Supplemental Table S3 lists the 32 features of myoepithelial nuclei that were measured, both morphological (size and shape) and textural (based on grey-level co-occurrence matrix, GLCM)^27^.

### HE stained image analysis

We analyzed HE stained slides which were serial sections of the p63 immunohistochemistry slides. Supplemental Figure S1 shows the flowchart of image analysis for HE stained image. All slides were scanned by the WSI scanner (Hamamatsu NanoZoomer 2.0-HT Slide Scanner) at 20x image magnification and are stored on a computer system at the same size and magnification as the p63 immunohistochemistry images. We selected 70 ROIs corresponding to those of the p63 immunohistochemistry images. For the HE image analysis, we applied analysis software made by NEC^21^,^28^ that segments the contour of cell nuclei. Note that segmentation on pathology slides is affected by color. In order to do accurate segmentation, we use a different segmentation method for p63 images. First, a trained classifier predicts the RGB color components of the hematoxylin based on the overall color histogram of the ROI. Then all pixels are projected onto the hematoxylin color vector resulting in a grey-level pixel map. Three types of filters are then used to locate nuclei centers. For small and mid-sized dense nuclei, two difference-of-Gaussian (DoG) filters were used, and for large nuclei, a Hough transform filter was used. Using these three filters, the analytical system produces three maps of hematoxylin peak signal which are then aggregated. After non-maxima suppression, polar cross sections were extracted from candidates’ centers. From peaks of Hematoxylin signal on the polar image, the system found the nucleus contour line using the snake line tracking method. As a result, all nuclei on the ROIs are segmented, including epithelial cells, stroma cells, lymphocytes, necrotic nuclei, etc. (Supplemental Figures S1–2). We then manually picked up the myoepithelial nuclei by referencing the corresponding p63 images (Supplemental Figures S1–3). Finally, we measured each identified cell using CellProfiler^26^ using the same features as the analysis of p63 immunohistochemistry images.

### Classification system

Classification was performed using Support Vector Machines (SVM)^29^ with Radial Basis Function (RBF) kernel using the LIBSVM^30^ package. First, we randomly split the data into a training (70%) and test set (30%). Next, we normalized the features across the training set (mean-centered, variance-scaled). The SVM hyper-parameters (C and γ of the RBF kernel) were calibrated using a grid search. We then computed the F-score (a measure of discrimination power) of each feature. Although there are correlations between some features, each has its own distinctive meaning rooted in the fields of biology and pathology. Using a feature selection process based on F-score metric^31^, we verified the usefulness of each features. On Supplemental Figure S2, we show that the combination of all 32 features provides the best accuracy. Finally, we applied the SVM model trained on the training dataset to the test data. This process was done 5 times with different training/test splits. In addition, we also split a total of 22 cases into 21 training cases and 1 test case randomly. This process was done 10 times with different training/test splits.

### Characterization of morphological heterogeneity based on SVM classification

To characterize the degree of heterogeneity of myoepithelial cells, we computed the spatial distribution of each type of myoepithelial cell classified by the SVM. Figure 1B shows an example of classified myoepithelial cell nuclei: dark blue nuclei shows myoepithelial cells classified as normal group, light blue nuclei as UDH group, light red nuclei as LG-DCIS group and dark red nuclei as HG-DCIS group. A bar graph shows the proportion of each classified cell type in the duct. Proportions of cell types are calculated both per duct and per patient.

### Electron microscopy

Fresh breast cancer tissue samples were put immediately into 2.5% glutaraldehyde (pH7.4) and fixed in 1% osmium tetroxide. These fixed samples were treated with a graded series of dehydration and then embedded in epon. Sections 0.1 μm thick were cut, stained with uranyl acetate and lead citrate, and examined with a transmission electron microscope at 60 kV. We observed a total of 584 cells.

### Protein interaction search in meta-analytic database

We searched the possible candidate crosstalk molecules using meta-analytic databases: the Human Protein Atlas Database (http://www.proteinatlas.org/)^32^ and Human Plasma Membrane Receptome database (http://receptome.stanford.edu/hpmr/home.asp)^33^. First, we searched 1879 proteins which included the key word “receptor” and are expressed on the normal myoepithelial cells in breast. Second, we narrowed down the list to 66 proteins which are predicted to be located on the plasma membrane. Third, we searched the corresponding ligands expressed on the breast luminal cells in Human Plasma Membrane Receptome database. As a result, 27 receptors/ligands couples were selected. Finally, we got 9 receptor/ligand couples after the exclusion of those with low levels of receptor/ligand expression (Supplemental Table S4).

### Survival analysis on meta-analytic database

To analyze the prognostic value of the extracted 9 receptor/ligand couples, we applied the Kaplan Meier (KM) plotter^34^ (http://kmplot.com/breast). The two groups, high and low ligand expression of cancer cells, could be compared in terms of relapse-free survival. The KM plotter is capable to assess the effect of 22,277 genes on survival in over 3000 breast cancer patients. The background databases were manually curated. Gene expression data and relapse free survival time were downloaded from GEO (Affymetrix microarrays only), EGA and TCGA. The database was handled by a PostgreSQL server, which integrated gene expression and clinical data simultaneously. The two patient cohorts were compared by a Kaplan-Meier survival plot, and the hazard ratio with 95% confidence intervals and log rank p value were calculated.

## Results

### Discriminant analysis by Support Vector Machine

We measured 32 morphological features of 11661 myoepithelial cell nuclei on pathology slides by computerized image processing: 4038 nuclei on normal cases, 3380 nuclei on UDH cases, 2485 on LG-DCIS cases and 1758 nuclei on HG-DCIS cases. By comparing the software segmentation results with the manually counted (pathologists) results in 20 ROIs, we evaluated segmentation accuracy. The average segmentation accuracy is 91% in p63 immunohistochemistry images and 85% in HE stained images. The morphological analysis resulted in a large matrix of morphological features (fundamental statistics are shown in Supplemental Figure S3). Tables 1 and 2 shows the confusion matrix of the SVM classifier for the task of classifying single cells into Normal/Benign and DCIS. Its accuracy was 75.2% for p63 immunohistochemistry images and 77.1% for HE stained images. For the task of classifying into four groups (Normal, UDH, LG-DCIS and HG-DCIS), the accuracy dropped to 53.2% (for p63 immunohistochemistry images) and 48.9% (for HE stained images). Figure 2A shows the comparison between the contribution level of each feature in p63 immunohistochemistry images and HE stained images. In both types, features that are directly related to nuclei flatness are the most discriminant: Minor Axis Length, Minor Feret’s Diameter and Ratio of the Minor/Major axis length. Interestingly, several features related to the contrast of nuclear texture in p63 immunohistochemistry images show higher contribution level than those in HE slide images: Contrast, DifferenceEntropy and InverseDifferenceMoment (Homogeneity). Figure 2B shows fundamental statistics of top 5 features based on their contribution level. These results show that the nuclei of myoepithelial cells in DCIS cases were more flat and spindle-like (similar to fibroblasts in interstitial tissue) than in Normal tissues. Furthermore, the contrast of nuclear staining in DCIS cases on p63 immunohistochemistry images was higher than in Normal cases, while their homogeneity (InverseDifferenceMoment feature) was lower.

### Morphological heterogeneity analysis of myoepithelial cells

Myoepithelial cells in lesions may be at various stages of neoplastic change, resulting in a heterogeneous distribution of their histologic types within a duct/ROI. In Fig. 3 and Supplemental Figure S4, we can observe that myoepithelial cells that were classified by the single-cell SVM classifier into different groups coexist in a duct. Nevertheless, for training the SVM classifier, we labeled all cells within a duct/ROI with the duct/ROI label, thus avoiding labeling individual cells. This has the advantage of avoiding the introduction of biases due to manual labeling. However, it results in noisy labels which may confuse the training procedure of the classifier and lower the accuracy results on the evaluation set. Notably, SVM classifiers have been shown to be fairly tolerant to label noise^35^. Hence, although the evaluation results reported above may appear low because of the noisy labels, the classifier has still learned a useful discriminative function and can successfully classify the nuclei into classes. To evaluate its true performance, we need to aggregate all nuclei classifications for a duct/ROI and use them to classify the duct/ROI. We calculated the proportion of cells in the four groups for each duct/ROI and for each patient (Fig. 4). We then applied a simple weighted majority decision method to classify each duct and patient into the four groups. With this approach we could obtain classifiers with significantly higher accuracies. For the duct classifier, the accuracy was 72.9% (p63) and 65.2% (HE) (Tables 3 and 4). For the patient classifier, the accuracy reached 90.9% (p63) and 81.8% (HE) (Tables 5 and 6). Furthermore, we also split a total of 22 cases (p63) into 21 training cases and 1 test case randomly, and evaluated the diagnostic prediction accuracy (Table 7). For the patient classifier in the task of classifying into Normal/Benign and DCIS, the accuracy also reached 90%.

### Electronic microscopic findings

To observe at higher resolution images of typical myoepithelial cells nuclei in benign and DCIS lesions, we used electron microscopy images. Figure 5 shows examples of myoepithelial cell nuclei. A typical myoepithelial cell in a benign duct has a larger and rounder nucleus (Fig. 5A), while those in DCIS duct were more flat (Fig. 5B). Additionally, organelles of these myoepithelial cells in DCIS were deformed and their boundaries unclear. Furthermore, basal laminas of DCIS ducts were monolayer (Fig. 5D), while those of benign ducts were made of multiple layers, similar to a Baumkuchen (Fig. 5C).

### Meta-analytic database analysis

We searched possible candidate molecules of crosstalk between luminal cells and myoepithelial cells in two databases: the Human Protein Atlas Database^32^ and the Human Plasma Membrane Receptome database^33^. We found 9 receptor/ligand couples which clearly express on both luminal cells and myoepithelial cells (Supplemental Table S4). We analyzed the Kaplan-Meier survival plot of large meta-analytic data. We found that breast cancers with low expression of the crosstalk molecules, Sonic Hedgehog (SHH) or Slit homolog 2 (SLIT2), have significantly shorter relapse-free survivals than those with high expression (Supplemental Figure S5).

## Discussion

Breast myoepithelial cells play an important role as physical barrier to cancer cell invasion and in producing the basement membrane. Gene expression of these cells changes dramatically between normal and DCIS tissues. Recent progress in digital pathology and image analysis has made it possible to quantitatively analyze large numbers of cells on tissue sections. In this study we aim to classify histological types of intraductal proliferative lesions using only the morphological and textural characteristics of myoepithelial cell nuclei and without any information about neoplastic cells. By training an SVM to classify myoepithelial cell nuclei into four groups and calculating their proportions within ducts and patients we demonstrated a high level of accuracy, above 90%. In both p63 immunohistochemistry images and HE stained images, myoepithelial nuclei in DCIS lesions showed flat or spindle-like morphology compared to benign lesions, and the contrast of nuclear staining of p63 immunohistochemistry in DCIS lesions was higher than in normal. Through electron microscopy examination, we found that typical myoepithelial cells in DCIS contain deformed and unclear organelles. Furthermore, basal laminas of DCIS ducts were monolayer while those of benign ducts were generally multilayer. These observations suggested that myoepithelial cells in DCIS may be less active than in benign lesion.

**Figure 6:**
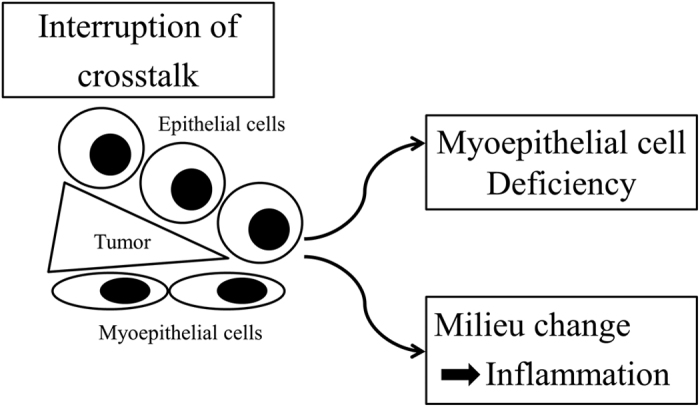
DCIS progression model. The spatial disruption of crosstalk between myoepithelial and epithelial cells through a wedge like protruding tongue of tumor cells, leads, in the case of SHH, towards myoepithelial cell deficiency and tumor cell dissemination, and, in the case of SLIT2, to a worsening of the local milieu, both having consequences for patient survival.

Why does the morphology of myoepithelial cell nuclei change during DCIS progression ? Both DCIS and benign proliferative lesions fill ducts with neoplastic and hyper-proliferative luminal cells which exert pressure on myoepithelial cells. However, only in DCIS were the nuclei of myoepithelial cells flattened. Therefore, the characteristic morphological change of myoepithelial nuclei in DCIS is not explained only by the physical pressure exercised by neoplastic luminal cells filling the ducts. Meanwhile, Fig. 3E showed that, while neoplastic cells push through between normal luminal cells and myoepithelial cells at the edge of a DCIS lesion like a wedge, the physical contact between both cell types is disrupted. Hence, we hypothesize that the local biochemical effect of neoplastic cells and the interruption of crosstalk between neoplastic cells and myoepithelial cells may lead to myoepithelial flatness, a lack of function and a lack of basal lamina synthesis. To verify our hypothesis, we searched possible candidate molecules of crosstalk between luminal cells and myoepithelial cells in two databases. Analyzing databases for paracrine signaling through receptor-ligand pairs, occurring both on luminal and myoepithelial cells, we found that breast cancers with low expression of the crosstalk molecules SHH and SLIT2 resulted in significantly shorter relapse-free survivals (Supplemental Figure S5). SHH is a ligand of the Hedgehog signaling pathway. It is a proto-oncogenic factor, which stimulates cancer cell proliferation in an autocrine manner through up-regulation of its expression^36^,^37^. Molecular inhibitors, such as Vismodegib, a Hedgehog signaling antagonist, have been used for therapeutic SHH down-regulation. However, it has been shown by Hu *et al*.^38^ that SHH down-regulation resulted in the progression of invasive cancer cells due to the loss of myoepithelial cells in DCIS. Therefore, our results suggest focusing on fine-tuning the paracrine cross-talk between luminal and myoepithelial cells, avoiding excessive SHH down-regulation when using molecular inhibitors such as Vismodegib. With regard to SLIT2, it is known that crosstalk between epithelial cells and myoepithelial cells using SLIT2/ROBO1 plays an important role in mammary gland morphogenesis^39^,^40^. SLIT2 limits basal cell proliferation and restricts mammary branching in morphogenesis. In turn, loss of SLIT2 reactivates the developmental program of branching, leading to invasive cancer. Further disruption of SLIT2 and ROBO1 has been shown to induce SDF1 and CXCR4 shifting the tumor microenvironment in an increased inflammatory state and further promoting invasion^41^. Thus, the spatial disruption of myoepithelial and epithelial cells observed here leads in the case of SHH deregulation towards myoepithelial cell deficiency and tumor cell dissemination, and, in the case of SLIT2 deregulation, to a worsening of the local milieu, both having consequences for patient survival (Fig. 6).

Clinically, the presence of myoepithelial cells is one of the major diagnostic criteria for pathologists to discriminate DCIS from invasive carcinomas. Furthermore, the number of myoepithelial cells can be helpful in distinguishing between benign proliferative breast disease and carcinoma on fine needle aspiration cytology smears^42^. Our results suggest that not only the number of myoepithelial cells but also the morphology of myoepithelial cell nuclei can be helpful in distinguishing between benign proliferative lesions and DCIS, especially by means of future computer assisted diagnosis (CAD) systems. So far, quantitative morphological analysis methods have been mostly limited to cultured cells with fluorescence staining that have high contrast contour lines. The reliable morphological quantification of cells is a complex visual challenge which can only be reasonably performed by computational image processing. As a result, digital pathology research including such quantitative morphological analysis has required advanced, specialized and often costly software. Recently, freeware image analysis programs that can segment cells on pathological slides have become available, including Ilastik^25^ which was used in this study. The combination of molecular based analysis and quantitative morphological analysis can provide useful tools for researchers to understand biological systems more deeply.

In summary, we firstly showed that breast intraductal proliferative lesions can be classified using quantitative morphological information of myoepithelial cells only, without any information about the epithelial tumor cells by quantitative morphological analysis and machine learning. We showed that in DCIS lesions myoepithelial cell nuclei showed a computationally recognizable, flattened morphology. Electron microscopy observations suggested that the activity of these cells was low. Further, we developed a paracrine cross-talk-based biological mechanism of DCIS progressing to invasive cancer. Our observations support novel approaches in clinical computational diagnostics as well as in therapy development against progression.

## Additional Information

**How to cite this article**: Yamamoto, Y. *et al*. Quantitative diagnosis of breast tumors by morphometric classification of microenvironmental myoepithelial cells using a machine learning approach. *Sci. Rep.* **7**, 46732; doi: 10.1038/srep46732 (2017).

**Publisher's note:** Springer Nature remains neutral with regard to jurisdictional claims in published maps and institutional affiliations.

## Supplementary Material

Supplementary Information

## Figures and Tables

**Table 1:** The confusion matrix on cell level p63 immunohistochemistry images.

Prediction				
Actual	Normal	UDH	LG-DCIS	HG-DCIS
Normal	**625**	150	31	18
UDH	265	**330**	84	13
LG-DCIS	115	103	**293**	34
HG-DCIS	145	91	84	**40**

4 groups: Accuracy 53.2% (kappa index 0.34). Normal/Benign vs DCIS: Accuracy 75.2% (kappa index 0.43).

**Table 2:** The confusion matrix on cell level Hematoxylin and Eosin stained images.

Prediction				
Actual	Normal	UDH	LG-DCIS	HG-DCIS
Normal	**263**	85	30	8
UDH	149	**133**	34	5
LG-DCIS	57	24	**110**	8
HG-DCIS	52	35	61	**18**

4 groups: Accuracy 48.9% (kappa index 0.27). Normal/Benign vs DCIS: Accuracy 77.1% (kappa index 0.46).

**Table 3:** The confusion matrix on duct level p63 immunohistochemistry images.

Prediction				
Actual	Normal	UDH	LG-DCIS	HG-DCIS
Normal	**13**	0	0	1
UDH	1	**12**	2	1
LG-DCIS	0	3	**13**	5
HG-DCIS	2	2	2	**13**

4 groups: Accuracy 72.9% (kappa index 0.64). Normal/Benign vs DCIS: Accuracy 84.3% (kappa index 0.68).

**Table 4:** The confusion matrix on duct level Hematoxylin and Eosin stained images.

Prediction				
Actual	Normal	UDH	LG-DCIS	HG-DCIS
Normal	**9**	4	0	1
UDH	2	**8**	2	3
LG-DCIS	1	1	**18**	1
HG-DCIS	0	2	7	**10**

4 groups: Accuracy 65.2% (kappa index 0.53). Normal/Benign vs DCIS: Accuracy 85.5% (kappa index 0.70).

**Table 5:** The confusion matrix on patient level p63 immunohistochemistry images.

Prediction				
Actual	Normal	UDH	LG-DCIS	HG-DCIS
Normal	**7**	0	0	0
UDH	0	**5**	0	0
LG-DCIS	0	0	**4**	1
HG-DCIS	0	1	0	**4**

4 groups: Accuracy 90.9% (kappa index 0.88). Normal/Benign vs DCIS: Accuracy 95.5% (kappa index 0.91).

**Table 6:** The confusion matrix on patient level Hematoxylin and Eosin stained images.

Prediction				
Actual	Normal	UDH	LG-DCIS	HG-DCIS
Normal	**5**	1	0	1
UDH	0	**4**	1	0
LG-DCIS	0	0	**5**	0
HG-DCIS	0	0	1	**4**

4 groups: Accuracy 81.8% (kappa index 0.76). Normal/Benign vs DCIS: Accuracy 90.9% (kappa index 0.82).

**Table 7:** Diagnostic prediction accuracy.

Case No.		Case. 1	Case. 2	Case. 3	Case. 4	Case. 5	Case. 6	Case. 7	Case. 8	Case. 9	Case. 10	Accuracy
Type		Normal	Normal	Normal	Normal	Normal	UDH	DCIS G1	DCIS G1	DCIS G3	DCIS G3	Total
**Cell Level**	4 groups	51%	49%	58%	42%	35%	42%	32%	20%	36%	37%	40%
	2 groups	82%	70%	70%	64%	67%	57%	89%	84%	46%	62%	69%
**Duct Level**	4 groups	2/2	2/2	2/2	2/2	1/2	3/3	2/4	2/7	3/5	3/3	69%
	2 groups	2/2	2/2	2/2	2/2	1/2	3/3	4/4	7/7	3/5	3/3	91%
**Case Level**	4 groups	○	○	○	○	×	○	○	×	○	○	**80%**
	2 groups	○	○	○	○	×	○	○	○	○	○	**90%**

*2 groups: Normal/Benign vs DCIS.
