# Supplementary material for: Quantitative diagnosis of breast tumors by morphometric classification of microenvironmental myoepithelial cells using a machine learning approach
Source: Sci Rep. 2017 Apr 25;7:46732. doi: 10.1038/srep46732 (PMC5404264; doi:10.1038/srep46732)

Supplementary Materials

Quantitative diagnosis of breast tumors by morphometric classification of microenvironmental myoepithelial cells using a machine learning approach

Yoichiro Yamamoto, § [1,2,3,4] Akira Saito, § [4,5,6] Ayako Tateishi,[1] Hisashi Shimojo,[1] Hiroyuki Kanno,[1] Shinichi Tsuchiya,[7] Ken-ichi Ito,[8] Eric Cosatto,[9] Hans Peter Graf, [9] Rodrigo R. Moraleda,[10,11] Roland Eils [2,3] and Niels Grabe [12,13]

§ Both authors contributed equally to this work

[1] Department of Pathology, Shinshu University School of Medicine, Nagano, Japan.

[2] Division of Theoretical Bioinformatics, German Cancer Research Center (DKFZ), Heidelberg, Germany.

[3] Department for Bioinformatics and Functional Genomics, Institute of Pharmacy and Molecular Biotechnology (IPMB) and Bioquant, University of Heidelberg, Heidelberg, Germany.

[4] RIKEN Center for Advanced Intelligence Project, Pathology Informatics Unit, Tokyo, Japan.

[5] Quantitative Pathology & Immunology, Tokyo Medical University, Shinjuku, Tokyo, Japan.

[6] Department of Molecular Pathology, Tokyo Medical University, Shinjuku, Tokyo, Japan.

[7] Diagnostic Pathology, Ritsuzankai Iida Hospital, Nagano, Japan.

[8] Division of Breast and Endocrine Surgery, Shinshu University School of Medicine, Nagano, Japan.

[9] Department of Machine Learning, NEC Laboratories America, NJ, USA.

[10] Applied Tumor Immunity Clinical Cooperation Unit, National Center for Tumor Diseases, German Cancer Research Center, Heidelberg, Germany.

[11] Department of informatics, Technical University Federico Santa Maria Valparaiso, Chile.

[12] Department of Medical Oncology, National Center for Tumor Diseases, University of Heidelberg, Heidelberg, Germany.

[13] Hamamatsu Tissue Imaging and Analysis Center, Bioquant, University of Heidelberg, Heidelberg, Germany.

Table S1. Characteristics of objects.

Total number of nuclei: 11661

	Cells (p63)	Cells (HE)	ROIs (p63)	ROIs (HE)	Cases	Age
Normal	2749	1289	14	14	7	65.4±13.9
UDH	2307	1073	16	16	5	56.6±7.6
LG-DCIS	1819	666	21	21	5	65.0±15.6
HG-DCIS	1203	555	19	19	5	63.8±11.6

(Mean±S.D.)

UDH: Usual ductal hyperplasia
LG-DCIS: Low Grade Ductal carcinoma in situ
HG-DCIS: High Grade Ductal carcinoma in situ

Table S2. Analyzed ROIs

	Total Cases	ROIs						
		Case1	Case2	Case3	Case4	Case5	Case6	Case7
Normal	**7**	2	2	2	2	2	2	2
UDH	**5**	4	2	3	3	4		
DCIS G1	**5**	4	7	4	4	2		
DCIS G3	**5**	4	3	5	3	4		

Table S3. Morphological features

Area & Shape

Area	Compactness	Eccentricity	Form Factor	Major Axis Length
Maximum Feret Diameter	Maximum Radius	Mean Radius	Median Radius	Minimum Feret Diameter
Minor Axis Length	Perimeter	Solidity	Ratio of Feret Diameter	Ratio of Axis Length
FracAtD 1of4*	FracAtD 2of4*	FracAtD 3of4*	FracAtD 4of4*	

Nuclear texture (GLCM)

Angular Second Moment	Contrast	Correlation	Difference Entropy	Difference Variance
Entropy	Info. Measure of Correlation1	Info. Measure of Correlation2	Inverse Difference Moment	Sum Average
Sum Entropy	Sum Variance	Variance		

* FracAtD: Fraction of total stain in an object at a given radius. The distribution is measured from the center of the object, where the center is defined as the point farthest from any edge. The numbering is from 1 (innermost) to 4 (outermost).

Table S4. Possible candidate molecules of crosstalk between luminal cells and myoepithelial cells.

Ligand (Luminal cell)	Receptor (Myoepithelial cell)
ADAM15, ADAM17, COL18A1	ITGB1
CDH1	ITGB7
DUSP18	ITGA3
DUSP18	ITGA7
FN1	SDC2
IL15	IL15RA
**SLIT2**	**ROBO1**
**SHH**	**SMO**
WNT7B	FZD1

Figure S1

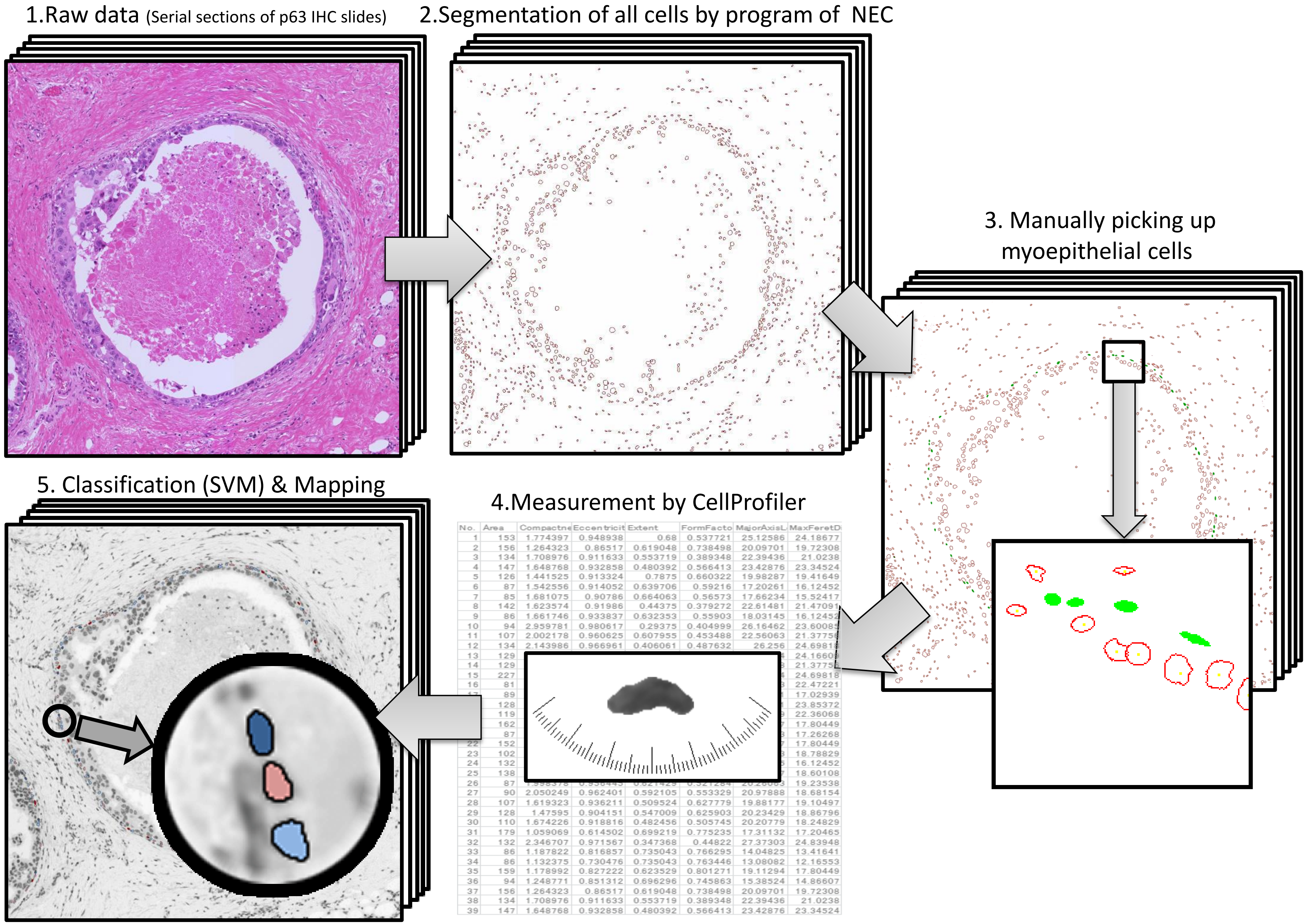
1.Raw data (Serial sections of p63 IHC slides)
2.Segmentation of all cells by program of NEC
3. Manually picking up myoepithelial cells
4.Measurement by CellProfiler
5. Classification (SVM) & Mapping
No.
Area
Compactne
Eccentricit
Extent
FormFacto
MajorAxisL
MaxFeretD

Figure S2

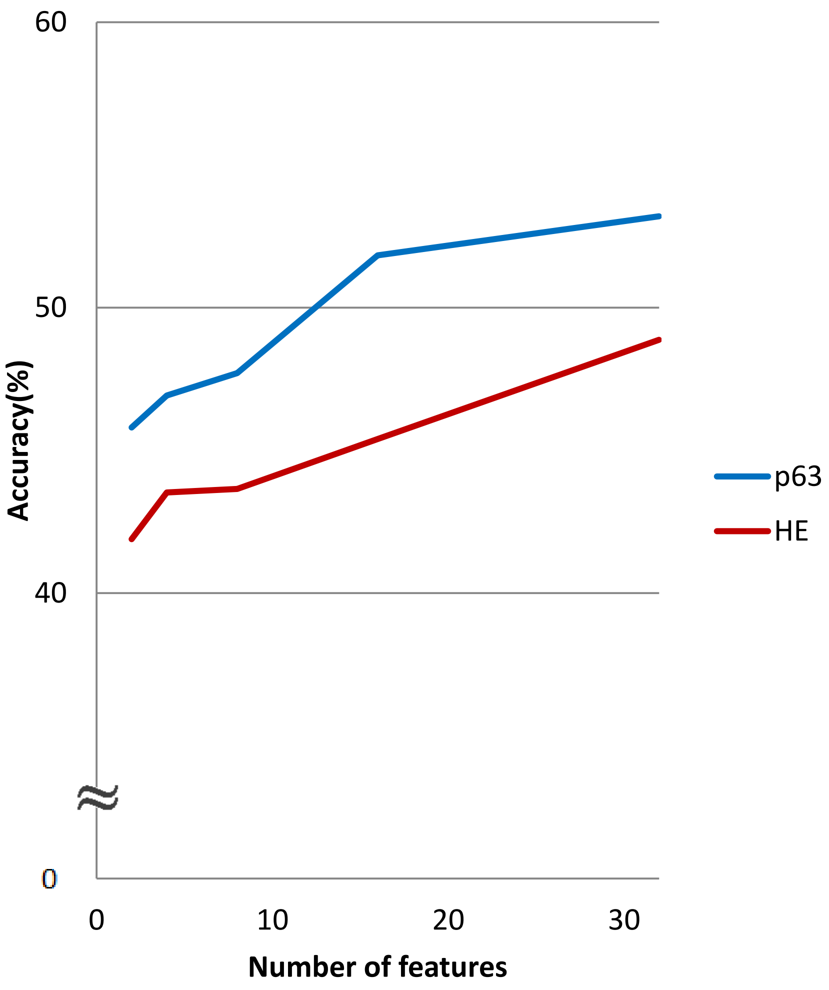

Figure S3

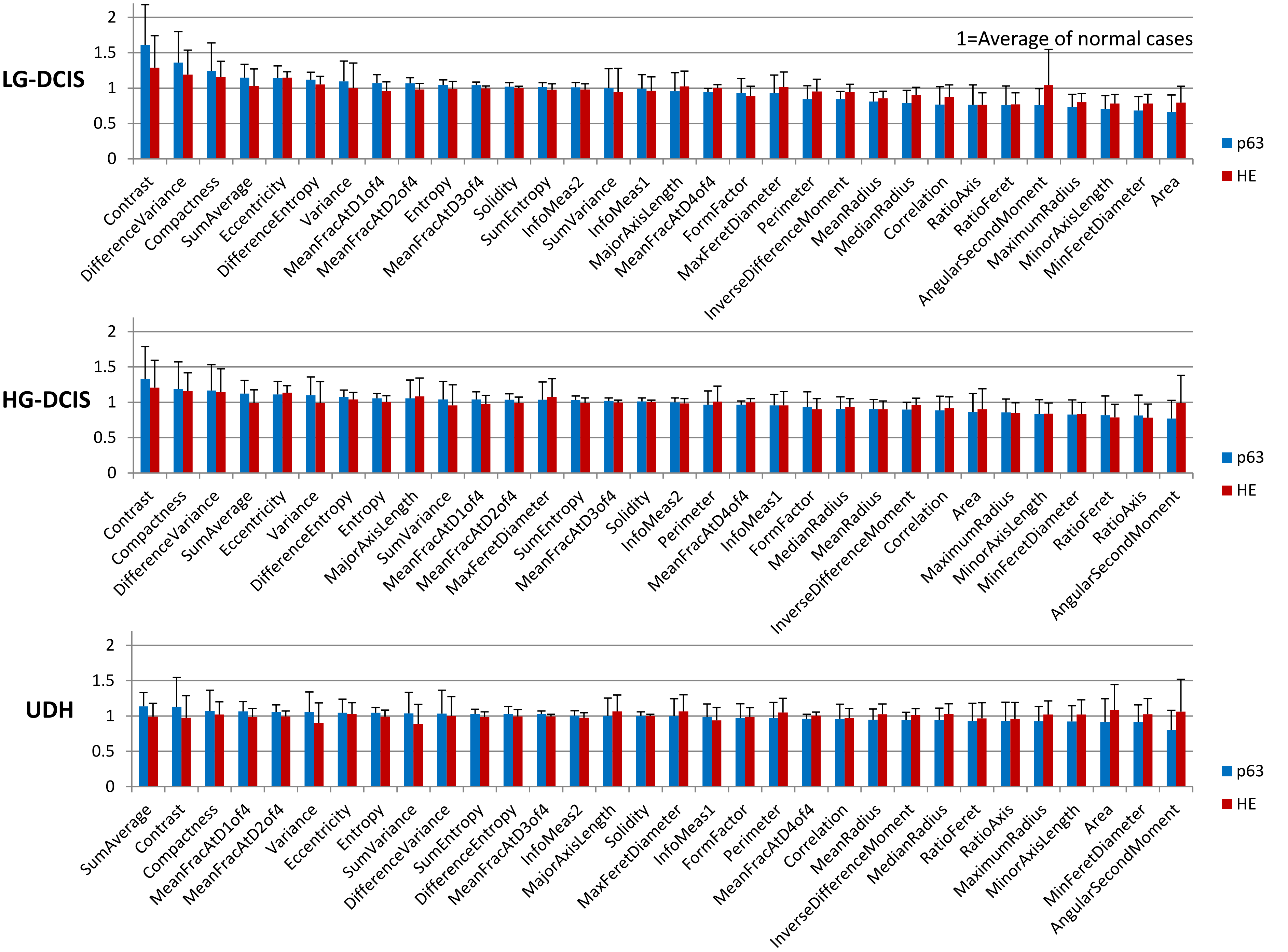
1=Average of normal cases
LG-DCIS
HG-DCIS
UDH
p63
HE
Contrast
DifferenceVariance
Compactness
SumAverage
Eccentricity
DifferenceEntropy
Variance
MeanFracAtD1of4
MeanFracAtD2of4
Entropy
MeanFracAtD3of4
Solidity
SumEntropy
InfoMeas2
SumVariance
InfoMeas1
MajorAxisLength
MeanFracAtD4of4
FormFactor
MaxFeretDiameter
Perimeter
InverseDifferenceMoment
MeanRadius
MedianRadius
Correlation
RatioAxis
RatioFeret
AngularSecondMoment
MaximumRadius
MinorAxisLength
MinFeretDiameter
Area

Figure S4

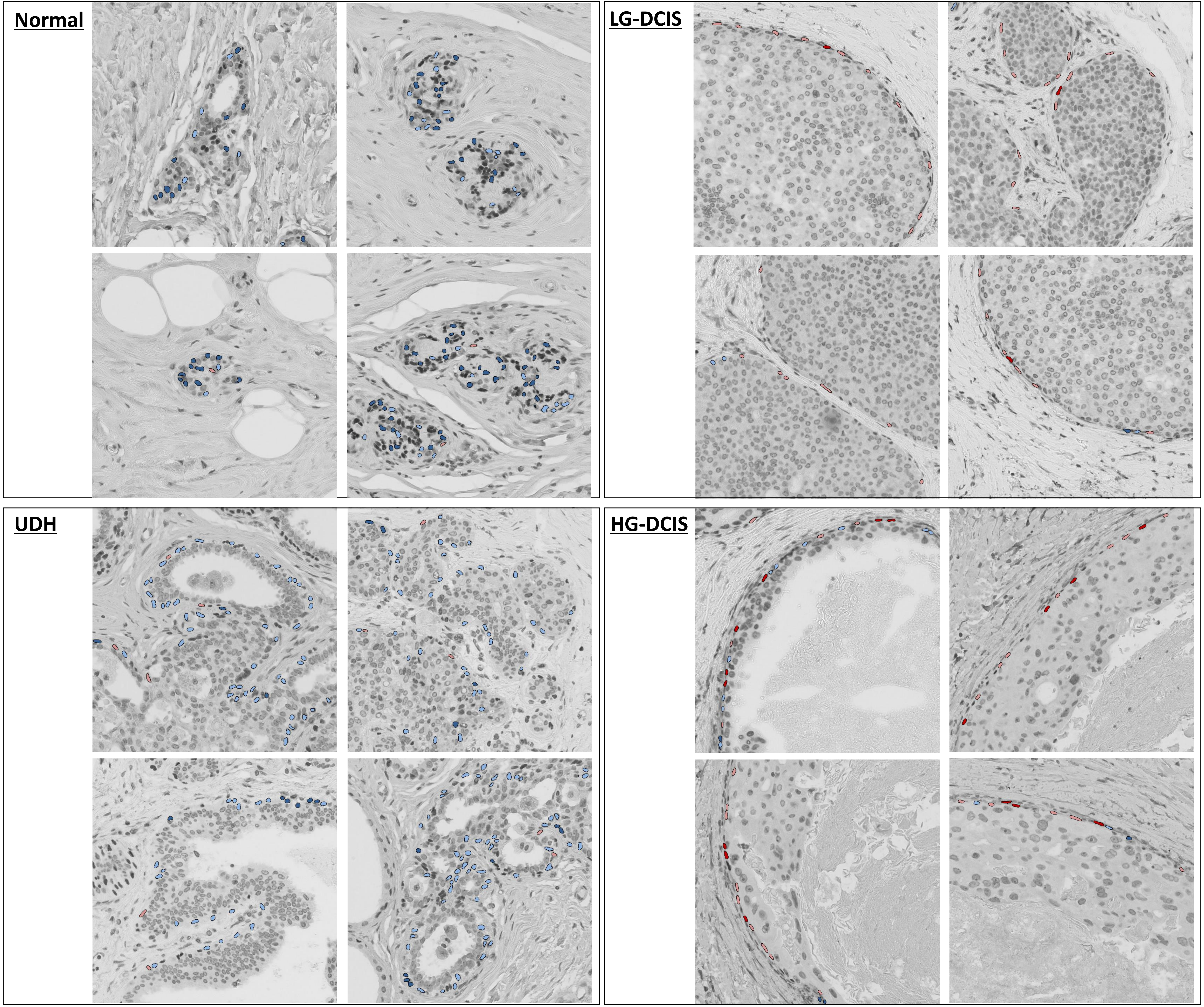

Figure S5

A

SHH

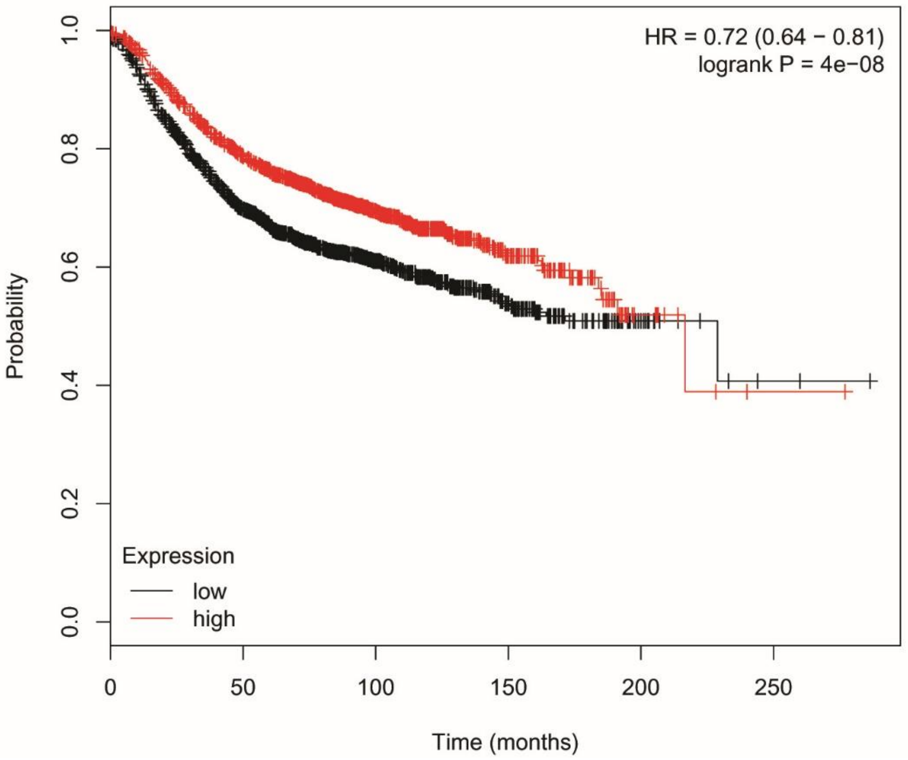

B

SLIT2

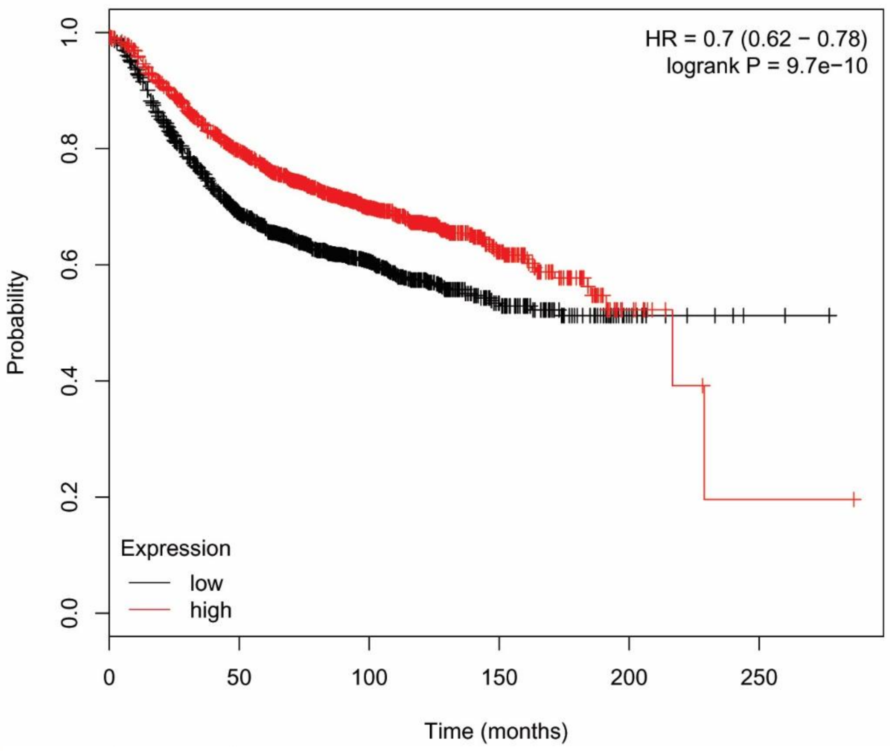

Supplement: Supplementary Information [file srep46732-s1.pdf]
